# Alpha-2 agonists for sedation in mechanically ventilated neurocritical care patients: a systematic review protocol

**DOI:** 10.1186/s13643-016-0331-4

**Published:** 2016-09-08

**Authors:** Alexandre Tran, Henrietta Blinder, Brian Hutton, Shane English

**Affiliations:** 1 Department of Epidemiology, University of Ottawa, Ottawa, Ontario Canada; 2 Department of General Surgery, University of Ottawa, Ottawa, Ontario Canada; 3 Clinical Epidemiology Program (CEP) Ottawa Health Research Institute, Ottawa, Ontario Canada; 4 Department of Medicine (Critical Care), University of Ottawa, Ottawa, Ontario Canada; 5 Department of General Surgery, The Ottawa Hospital, Ottawa, Ontario Canada; 6 The Ottawa Hospital Civic Campus, Loeb Research Building, Main Floor 725 Parkdale Avenue, Office WM150E, Ottawa, Ontario K1Y 4E9 Canada

**Keywords:** Alpha-2 agonists, Dexmedetomidine, Clonidine, Sedation, Neurocritical, Mechanical ventilation

## Abstract

**Background:**

Sedation is an important consideration in the care of the neurocritically ill patient. It provides anxiety and relief, facilitates procedures and nursing tasks, and minimizes intolerance of mechanical ventilation. Alpha-2 agonists such as dexmedetomidine and clonidine have been shown to be an effective alternative in the general critical care population by reducing duration of mechanical ventilation and length of stay in the intensive care unit (ICU), as compared to traditional sedative agents such as propofol or benzodiazepines. However, there is a paucity of literature detailing their utility and safety in neurocritical care, a population that presents unique considerations for management of global and cerebral hemodynamics, agitation, and facilitation of neurological assessments. The objective of this review is to assess the efficacy and safety of alpha-2 agonists for non-procedural sedation in mechanically ventilated brain-injured patients.

**Methods:**

We will search the Embase and MEDLINE databases for all randomized controlled trials, prospective and retrospective cohort studies examining neurocritically ill adult patients aged 18 years and older who are on mechanical ventilation and receiving alpha-2 agonists for non-procedural sedation. Primary outcomes of interest include effect on mean arterial pressure (MAP), intracranial pressure (ICP), and cerebral perfusion pressure (CPP). Secondary outcomes include adverse events, duration of mechanical ventilation, 30-day mortality, ICU length of stay, incidence of delirium, and quality of sedation. Continuous outcomes will be presented as means and mean differences and discrete counting events will be presented as event rates. Pre-defined criteria for heterogeneity are provided for determination of pooling eligibility. Where appropriate, we will pool estimates for individual outcomes. Planned subgroup analyses include specific alpha-2 agonist agent, study design, clinical diagnosis, dosing regimen, and use of adjunctive agents. Quality of evidence for the recommendation will be assessed using the Grading of Recommendations, Assessment, Development and Evaluation (GRADE) approach where appropriate.

**Discussion:**

This systematic review will summarize the evidence on the efficacy and safety for the use of alpha-2 agonists as sedative agents in the neurocritical care population.

**Systematic review registration:**

PROSPERO CRD42016037045

**Electronic supplementary material:**

The online version of this article (doi:10.1186/s13643-016-0331-4) contains supplementary material, which is available to authorized users.

## Background

### Description of the condition

Sedation is an important component in the care of critically ill patients requiring mechanical ventilation. Goals of sedation in medical and surgical intensive care units (ICU) commonly include reductions in pain, anxiety, catecholamine activity and oxygen demand [1], facilitation of procedures or nursing care, and minimization of ventilator dyssynchrony [2].

Sedation for the critically ill patient presents an array of challenges and adverse consequences. It is associated with the development of delirium, which in turn may lead to prolonged hospitalization and worse patient outcomes [3]. The challenging and unpredictable pharmacokinetics and pharmacodynamics of the critically ill patient may result in drug accumulation, prolonged drug effect, and sleep disturbances. Oversedation of patients leads to prolonged weaning from ventilation, unreliable neurologic and delirium screening assessments, and increased risk of post-traumatic stress disorder after discharge [4]. In addition, many classes of sedative agents may cause adverse hemodynamic events including hypotension and arrhythmias [5].

ICU populations are very heterogeneous groups of patients. Head injury is a common cause for ICU admission and is often accompanied with severe rates of mortality and morbidity [6]. In the specific ICU patient sub-population with significant intra-cranial pathology, the intensivist faces a rather unique set of considerations when selecting sedative agents. These include the need for optimization of intracranial pressure (ICP), cerebral perfusion pressure (CPP), and cerebral oxygen consumption, and the management of neurogenic hyperventilation and treatment of aggressive agitation or delirium [7]. The intensivist must manage a number of factors to which the neurocritical patient is uniquely sensitive, such as blood pressure, oxygenation, carbon dioxide, temperature, and glucose levels in balance with the need for frequent and accurate neurologic assessments. Failure to appropriately address these considerations can potentially result in catastrophic outcomes.

Despite a wide selection of available drug classes, there is little consensus regarding an optimal agent or regimen. Systematic reviews of randomized controlled trials in medical and surgical ICUs evaluating commonly used agents such as propofol, midazolam, or other sedatives demonstrated conflicting results regarding weaning duration, length of stay, quality of sedation, and rates of adverse events [3, 8–12].

### Description of the intervention

Alpha-2 agonists, such as dexmedetomidine and clonidine, have gained recent popularity as sedative agents in neurocritical patients. They provide sedative, anxiolytic, and some analgesic effects without causing respiratory depression [2]. The properties of alpha-2 agonists allow for their effective use in procedural sedation and reduce the need for adjunctive anxiolytics or opioids [13]. In addition, there is a pre-clinical evidence to suggest a direct neuroprotective effect in traumatic brain injury models [14]. Within the intensive care setting, alpha-2 agonists have been demonstrated to reduce the duration of mechanical ventilation and ICU length of stay as compared to traditional sedatives such as propofol or benzodiazepines [2]. These improved outcomes could be due to better arousal, easier communication, retained spontaneous respiration, and reduction in delirium. Clonidine has historically been used quite commonly in adult ICUs despite a paucity of literature to evaluate its effectiveness [15]. Dexmedetomidine, an IV formulation introduced more recently, is known to be considerably more expensive than traditional sedatives, but offers considerable overall savings due to lower occurrence of delirium and shorter time to extubation [16, 17]. There is no previous literature comparing clonidine and dexmedetomidine directly.

### How the intervention might work?

Alpha-2 agonists bind to transmembrane G protein-binding adrenergic receptors in the periphery (alpha-2a subtype) as well as in the brain and spinal cord (alpha-2b and alpha-2c subtypes) [13]. The activation of these receptors leads to suppression of neuronal activity and inhibition of norepinephrine release, particularly in the locus coeruleus, the principal site for norepinephrine synthesis in the brain [18]. These agents therefore have profound effects on the modulation of anxiety, arousal, and sleep. In addition, alpha-2 agonists may present hemodynamic effects, resulting most commonly in hypotension and mild bradycardia via direct sympatholytic effects [5]. Clonidine, more so than dexmedetomidine, has been implicated as a cause of rebound hypertension following its discontinuation. These potential hemodynamic effects directly affect ICP, CPP, and the resultant cerebral blood flow.

### Why it is important to do this review?

The use of alpha-2 agonists for non-procedural sedation in critical care is a relatively novel concept and patterns of use vary worldwide. Certain jurisdictions (e.g., Europe) have had access to the IV formulation of alpha-2 agonists like clonidine for some time whereas this has only been possible in Canada more recently with the development of dexmedetomidine. A recent systematic review by the Cochrane Collaboration demonstrated that compared with traditional sedatives, dexmedetomidine shortened the duration of mechanical ventilation and ICU length of stay in the general critical care population [2]. There was no evidence for a difference in delirium or mortality. However, there was no specific analysis for the acute brain injury population or safety evaluation of key neurologic care indicators such as ICP or CPP. Another systematic review by Roberts et al. studied critically ill adults with severe traumatic brain injury but evaluated only traditional sedatives such as propofol, benzodiazepines, and opioid analgesics [12]. The management of the critically ill brain-injured patient requires frequent and reliable neurological assessment, as well as special consideration given to global and cerebral hemodynamics. Given these unique challenges, a comprehensive analysis is needed to assess the safety and efficacy of alpha-2 agonists for long-term sedation in this patient population.

### Objective

The objective of this review is to assess the efficacy and safety of alpha-2 agonists for non-procedural sedation in critically ill, mechanically ventilated brain-injured adult patients.

## Methods/design

This systematic review protocol was designed using the Preferred Reporting Items for Systematic Review and Meta-Analyses Protocol (PRISMA-P) checklist (see Additional file 1) [19, 20]. This protocol has been registered with the PROSPERO International Prospective Register of Systematic Reviews (PROSPERO # CRD42016037045). Any subsequent amendments made to the protocol during the review process will be clearly outlined and discussed in the review manuscript.

### Eligibility criteria

Studies will be selected according to the criteria outlined below. The criteria have been outlined according to the PICOS (Population, Intervention, Comparator, Outcomes, Study Design) framework.

### Population

We will include all studies examining neurocritically ill adult patients (age ≥18 years) who are on invasive mechanical ventilation and require non-procedural sedation. Neurocritically ill patients are defined as those admitted to an ICU with a primary neurological diagnosis, such as a stroke, hemorrhage, traumatic brain injury, or post-neurosurgical care. Studies with a mixed ICU population will be included if at least 50 % of the population is composed of neurocritically ill patients, or if a separate neurocritical care subgroup is clearly presented within the study. In instances of lack of clarity, clarification with the corresponding author will be attempted. Studies composed of mixed invasive and non-invasive ventilation patients will be included. Studies evaluating patients without neurological diagnoses or mechanical ventilation will be excluded.

### Intervention

The intervention of interest is the use of an alpha-2 agonist as an agent for non-procedural sedation. The alpha-2 agonist can be administered as a stand-alone or adjunctive agent. There is no limit on dose, frequency, or route of administration. There is no restriction for presence or type of sedation protocol. Studies evaluating the use of alpha-2 agonists solely for procedural sedation will be excluded.

### Comparators

The comparator of interest is any standard sedative regimen such as propofol, benzodiazepines, or opioids. This includes between group comparisons and within group comparisons (such as before-after designs). We anticipate a paucity of literature examining the safety and efficacy of alpha-2 agonists for non-procedural sedation in neurocritical care patients. As such, we will be as inclusive as possible and include studies without a comparator. No studies will be excluded for lack of or type of comparator.

### Outcomes

The primary goal of this review is to evaluate the safety and efficacy of these agents with regard to neurophysiologic parameters. Complex patient-centered outcomes are not expected to be available given the paucity of literature and may be a reasonable focus of future study once safety and efficacy is established. Therefore, the primary outcomes of interest are mean arterial pressure (MAP), intracranial pressure (ICP), and cerebral perfusion pressure (CPP). These outcomes will be described as means, within group mean differences, and across group mean differences (before and after administration).

These primary outcomes were chosen as they represent the principal physiologic parameters optimized in the management of the neurocritical care population. Changes in these parameters, related or not to sedative choice, can have a large impact on patient safety and outcome. Given that we anticipate a great deal of heterogeneity in hemodynamic parameters reported by studies, we will collect data on all parameters reported by authors to ensure this review is as inclusive as possible. No studies will be excluded based on the lack of reported outcomes.

Secondary outcomes include adverse events as defined by the study authors (including bradycardia, hypotension, or any other adverse event), duration of mechanical ventilation, incidence of delirium (as assessed by the Confusion Assessment Method—ICU), quality of sedation (Richmond Agitation–Sedation scale or COMFORT score), ICU length of stay, and 30-day mortality.

### Study design

We will include all completed clinical studies reporting the use of an alpha-2 agonist as a sedative in neurocritical care patients in our quantitative review, including randomized controlled trials, quasi-randomized trials, and retrospective and prospective cohort studies. There will be no date or language restrictions. In-progress studies identified from the clinicaltrials.gov registry and CENTRAL Cochrane will be included in a qualitative analysis. Letters to the editor, case reports, editorial reviews, and guidelines will be excluded.

#### Data sources

A comprehensive literature search will be conducted in MEDLINE and Embase using a pre-defined search strategy as detailed below. There will be no restrictions on language or time period. We will additionally hand-search the reference lists of all primary studies and previously published systematic reviews for relevant studies. The conference abstracts of the European Society of Intensive Care Medicine Congress, Neurocritical Care Society Annual Meeting, Pulmonary and Critical Care Medicine, and Critical Care Canada Forum for the last 3 years will be searched, and the first authors of relevant abstracts will be contacted for further information. To locate unpublished or ongoing trials, the clinicaltrials.gov registry and CENTRAL Cochrane Library will be searched as well.

#### Search strategy

A search strategy was developed under the guidance of a health information specialist with expertise in clinical research and was subsequently peer reviewed by a clinical expert. Keywords were derived based on the population of interest, neurocritical care patients, and the intervention, alpha-2 agonists. The final search strategy is comprised of both relevant Medical Subject Headings (MeSH) and synonyms identified from the keywords. The search strategies for MEDLINE and Embase are available in Appendices 1 and 2, respectively. MEDLINE and Embase will be searched through the Ovid interface.

#### Study selection process

Literature search results will be de-duplicated in Endnote software (version X7, Thomson Reuters) [21] and then uploaded to Covidence, a web-based systematic review tool that facilitates screening (online version, Alfred Health) [22]. The assessors will not be blinded to author, institution, or journal of publication. Titles and abstracts will be independently screened for inclusion by two reviewers (AT, HB), and disagreements will be resolved by a clinical expert (SE) if needed. If insufficient information is available from an abstract to determine eligibility, the study will be included for a full-text review. Full text reports will be independently assessed for eligibility using an approach to that described for review of abstracts. Reasons for excluding full-text articles will be documented. The entirety of the study selection process will be documented and presented using a flow diagram within the final review as recommended by the PRISMA statement [23].

#### Data collection and analysis

### Data extraction and management

Data extraction will be performed independently and in duplicate using a data collection form implemented in Microsoft Excel 2013 that will be piloted a priori. Abstracted data will include the following: publication characteristics (study title, first author, year of publication, country of study origin), key patient characteristics (age, sex, primary ICU diagnoses, and primary neurological diagnoses, injury severity/characteristics), intervention and comparator characteristics (agent, dose, route, sedation protocol, and adjunctive sedatives), outcome data (including means/standard deviation or median/interquartile and numbers of events and sample size for binary endpoints), and study methods for the risk of bias assessment described below. Disagreements in data extraction will be resolved by consensus or by a third reviewer with methodologic and clinical expertise as required.

### Assessing the risk of bias

The risk of bias in each included study will be assessed independently by two reviewers. Disagreements will be resolved by consensus or by a third reviewer with clinical expertise if required. These assessments will be completed for each individual outcome of interest. Risk of bias for randomized controlled trials will be assessed using the Cochrane Risk of Bias tool [24], which rates bias as low risk, high risk, or unclear risk. This assessment is based upon criteria which include sequence generation, allocation concealment, blinding, selective outcome reporting, and presence of other biases. The risk of bias for observational studies will be assessed using the Newcastle-Ottawa Quality Assessment scale [25]. This assessment, scored out of 9, is based on criteria such as cohort and control selection, comparability of the two groups as well as outcome assessment and follow-up. All studies will be included in this review regardless of their risk of bias.

### Dealing with missing data

We will attempt to contact the corresponding authors of studies with missing data up to two times via email. If missing data is not located, available data will be analyzed and the potential impact of the missing data will be discussed as a limitation.

### Data synthesis

Where pooling of outcome data is appropriate, we will use Comprehensive Meta-Analysis software (Version 3, Biostat Inc) [26] to perform meta-analyses using a random effects model. Effect sizes for each outcome will be determined using the inverse-variance method. The results will be presented as either mean differences with 95 % CI for continuous outcomes or event rates with 95 % CI for discrete counting events.

We anticipate considerable clinical heterogeneity in the literature with regard to study design, clinical diagnosis, and definition of the outcome measures. In order to address this concern, we will plan for the following subgroup analyses:

1. Specific alpha-2 agonist (dexmedetomidine, clonidine, other)
  - Evaluated for all primary and secondary outcomes
2. Clinical diagnosis (TBI, stroke, hemorrhage, other)
  - Evaluated for all primary and secondary outcomes
3. Dosing regimen (rapid dosing—IV bolus then infusion, slow dosing—oral route or IV infusion without bolus)
  - Evaluated for adverse events
4. Adjunctive agents (other co-sedatives or analgesics, none)
  - Evaluated for adverse events, risk of delirium, and quality of sedation

Statistical heterogeneity of study findings will be assessed using the Cochrane Q and the *I*^2^ statistic in order to determine appropriateness of data synthesis. The *I*^2^ statistic values of 25, 50, and 75 % correspond to low, moderate, and high heterogeneity, respectively. If there is demonstration of significant heterogeneity as defined by a *p* < 0.10 and an *I*^2^ statistic >75 %, then pooling of data will not be performed. In such cases, the results will be described qualitatively.

### Sensitivity analysis

A sensitivity analysis may be used to restrict analysis to randomized and prospective cohort study designs to assess the influence of data from other study designs which may be prone to increased bias. Post hoc sensitivity analyses will be performed where appropriate, including those informed by risk of bias assessments.

### Assessing the quality of evidence

The Grading of Recommendations Assessment, Development and Evaluation (GRADE) approach will be used to assess the quality of evidence for each outcome where appropriate, but the anticipated heterogeneity of study design may limit our ability to do so [27–32]. This approach describes the level of confidence for which an estimate of effect is close to the value of interest. The overall quality of evidence is summarized as high, moderate, low, or very low. The GRADE assessment is based on the following criteria: risk of bias and study limitations, directness, consistency of results, precision, publication bias, magnitude of effect, dose-response gradient, and residual confounding. A final quality of evidence grade and strength of recommendation (strong or weak) will be provided for or against the intervention under view.

#### Preparation of the completed review

The PRISMA statement and the meta-analysis of observational studies in epidemiology (MOOSE) checklist will be used when drafting a summary report of the completed review [23, 33].

## Discussion

While alpha-2 agonists such as dexmedetomidine and clonidine have been shown to be an effective class of sedative in the general critical care population, there is a paucity of literature detailing their utility and safety in neurocritical care. This systematic review will summarize the evidence on the efficacy and safety for the use of alpha-2 agonists as sedative agents in the neurocritical care population.

## Additional file

Additional file 1: PRISMA-P 2015 checklist.

## Appendix 1

### Search strategy for MEDLINE database

1 Adrenergic alpha-Agonists/

2 exp Adrenergic alpha-2 Receptor Agonists/

3 (alpha adj3 agonist*).tw.

4 clonidine.tw.

5 dexmedetomidine.tw.

6 precedex.tw.

7 exp Intracranial Hemorrhages/

8 exp Brain Injuries/

9 exp Stroke/

10 neurocritical.tw.

11 brain injur*.tw.

12 brain trauma*.tw.

13 head trauma*.tw.

14 head injur*.tw.

15 neurotrauma*.tw.

16 craniocerebral trauma*.tw.

17 craniocerebral injur*.tw.

18 neuro* injur*.tw.

19 neuro* trauma*.tw.

20 brain h?emorrhage*.tw.

21 subarachnoid h?emorrhage*.tw.

22 (cerebr* adj2 h?emorrhage*).tw.

23 intracerebr* h?emorrhage*.tw.

24 posterior fossa h?emorrhage*.tw.

25 intracranial h?emorrhage*.tw.

26 cerebral infarct*.tw.

27 stroke*.tw.

28 cerebrovascular accident*.tw.

29 brain vascular accident*.tw.

30 TBI.tw.

31 SAH.tw.

32 ICH.tw.

33 CVA.tw.

34 1 or 2 or 3 or 4 or 5 or 6

35 7 or 8 or 9 or 10 or 11 or 12 or 13 or 14 or 15 or 16 or 17 or 18 or 19 or 20 or 21 or 22 or 23 or 24 or 25 or 26 or 27 or 28 or 29 or 30 or 31 or 32 or 33

36 34 and 35

## Appendix 2

### Search strategy for Embase database

1 alpha adrenergic receptor stimulating agent/

2 alpha 2 adrenergic receptor stimulating agent/

3 clonidine/

4 dexmedetomidine/

5 (alpha adj3 agonist*).tw.

6 clonidine.tw.

7 dexmedetomidine.tw.

8 precedex.tw.

9 acquired brain injury/

10 diffuse axonal injury/

11 traumatic brain injury/

12 brain injury/

13 head injury/

14 exp cerebrovascular accident/

15 exp brain hemorrhage/

16 neurocritical.tw.

17 brain injur*.tw.

18 brain trauma*.tw.

19 head trauma*.tw.

20 head injur*.tw.

21 neurotrauma*.tw.

22 craniocerebral trauma*.tw.

23 craniocerebral injur*.tw.

24 neuro* injur*.tw.

25 neuro* trauma*.tw.

26 brain h?emorrhage*.tw.

27 subarachnoid h?emorrhage*.tw.

28 (cerebr* adj2 h?emorrhage*).tw.

29 intracerebr* h?emorrhage*.tw.

30 posterior fossa h?emorrhage*.tw.

31 intracranial h?emorrhage*.tw.

32 cerebral infarct*.tw.

33 stroke*.tw.

34 cerebrovascular accident*.tw.

35 brain vascular accident*.tw.

36 1 or 2 or 3 or 4 or 5 or 6 or 7 or 8

37 TBI.tw.

38 SAH.tw.

39 ICH.tw.

40 CVA.tw.

41 9 or 10 or 11 or 12 or 13 or 14 or 15 or 16 or 17 or 18 or 19 or 20 or 21 or 22 or 23 or 24 or 25 or 26 or 27 or 28 or 29 or 30 or 31 or 32 or 33 or 34 or 35 or 37 or 38 or 39 or 40

42 36 and 41

## Abbreviations

ICU: Intensive care unit
ICP: Intracranial pressure
MAP: Mean arterial pressure
CPP: Cerebral perfusion pressure
GRADE: Grading of Recommendations, Assessment, Development and Evaluation
PRISMA-P: Preferred Reporting Items for Systematic review and meta-analyses protocol
MOOSE: Meta-analysis of observational studies in epidemiology
